# The number of repeated observations needed to estimate the habitual physical activity of an individual to a given level of precision

**DOI:** 10.1371/journal.pone.0192117

**Published:** 2018-02-01

**Authors:** Patrick Bergman

**Affiliations:** Department of Sport Sciences, Linnaeus University, Kalmar, Sweden; Pennington Biomedical Research Center, UNITED STATES

## Abstract

Physical activity behavior varies naturally from day to day, from week to week and even across seasons. In order to assess the habitual level of physical activity of a person, the person must be monitored for long enough so that the level can be identified, taking into account this natural within-person variation. An important question, and one whose answer has implications for study- and survey design, epidemiological research and population surveillance, is, for how long does an individual need to be monitored before such a habitual level or pattern can be identified to a desired level of precision? The aim of this study was to estimate the number of repeated observations needed to identify the habitual physical activity behaviour of an individual to a given degree of precision. A convenience sample of 50 Swedish adults wore accelerometers during four consecutive weeks. The number of days needed to come within 5–50% of an individual's usual physical activity 95% of the time was calculated. To get an idea of the uncertainty of the estimates all statistical estimates were bootstrapped 2000 times. The mean number of days of measurement needed for the observation to, with 95% confidence, be within 20% of the habitual physical activity of an individual is highest for vigorous physical activity, for which 182 days are needed. For sedentary behaviour the equivalent number of days is 2.4. To capture 80% of the sample to within ±20% of their habitual level of physical activity, 3.4 days is needed if sedentary behavior is the outcome of interest, and 34.8 days for MVPA. The present study shows that for analyses requiring accurate data at the individual level a longer measurement collection period than the traditional 7-day protocol should be used. In addition, the amount of MVPA was negatively associated with the number of days required to identify the habitual physical activity level indicating that the least active are also those whose habitual physical activity level is the most difficult to identify. These results could have important implications for researchers whose aim is to analyse data on an individual level. Before recommendations regarding an appropriate monitoring protocol are updated, the present study should be replicated in different populations.

## Background

A large body of evidence exists that shows the benefits of physical activity and the negative consequences of sedentary behaviour for physical and mental health [1–3]. The accurate measurement of habitual physical activity is important in order to understand the relationship between frequency, duration and amount of physical activity and health. A central property of physical activity behaviour is that in free-living populations, it naturally varies from day-to-day around a true mean level of physical activity. This true mean level of physical activity is often referred to as the habitual level of physical activity of an individual and in this study is defined, slightly modified from Lui's version for diet, as: "the hypothetical average around which that individual's physical activity varies" [4]. In the short term, this variation may be influenced by such things as the weather [5] or what day of the week it is [6] and over longer periods of time, seasonal variations [7] in physical activity are seen.

The natural variation of physical activity implies that, in order to assess the habitual level of physical activity of a group or an individual, that group or individual must be monitored for long enough so that the habitual level of physical activity of the group or individual can be identified. The field of nutritional epidemiology (which shares many of the methodological problems with physical activity research when it comes to measuring exposure) has described four different levels of measurement precision needed to answer different types of research questions [4, 8]. Level 1 is the accuracy needed to be able to determine the mean level of physical activity in a group, such as when estimating the prevalence in large populations or following trends over time. Level 2 is the accuracy needed to describe the mean and distribution of physical activity in a group i.e. being able to make comparisons between groups. Level 3 is the accuracy required in order to rank individuals in a group from the most active to the least active, used quite often in epidemiology to create heterogenous groups (differing in level of exposure). However, sometimes information on an individual’s absolute level of physical activity, rather than their relative (rank) level, is needed. For example, in order to measure effects of interventions, such as counseling or “physical activity on prescription”, or to perform analysis correlating a biomarker measured on individual level with the activity level of the same individual; this is level 4.

Depending on the level of precision that is needed, there are two separate ways of increasing the precision of the measurement; either by increasing the number of subjects in a study, or by increasing the number of repeated observations for each subject. For studies aiming to answer research questions requiring measurements at level 1 or level 2, increasing the number of subjects, rather than the number of repeated observations within each subject, is adequate. It can be assumed that the random noise introduced by the day-to-day variation is cancelled by subjects that are either more active or less active compared to their habitual activity level and that on average the group mean remains unchanged [9]. For studies related to measurements at level 3 or level 4, increasing the number of observations within each subject is required. However, within physical activity research it appears as if there has been a long-standing misconception that data on level 3 provides information on the habitual (usual, typical) level of physical activity of an individual, e.g. [10–22]. Estimating the number of days required to satisfy level 3 assumptions, i.e. the rank order of the individuals in a group, is normally done by first calculating an intra-class-correlation coefficient (ICC) and then entering the ICC into the Spearman-Brown prophecy formula. But this is not sufficient to determine the habitual physical activity of an individual.

Consider the following situation. The ICC can be calculated as $ICC=\frac{\sigma_{b}^{2}}{\sigma_{b}^{2}+\sigma_{w}^{2}}$ where $\sigma_{b}^{2}$ is the between-subject variation and $\sigma_{w}^{2}$ is the within-subject variation. If ${\;\sigma }_{b}^{2}=100$ and $\sigma_{w}^{2}=25$ then the ICC = 0.8. However, if ${\;\sigma }_{b}^{2}=10$ and $\sigma_{w}^{2}=2.5$ then the ICC is still 0.8 even if the within-subject variation differs by a factor of 10.

Given that the pattern of habitual level of physical activity in an individual is estimated by the within-subject variation [4, 8] there is a lack of information regarding the number of repeated observations required to achieve level 4 of accuracy. Particularly as most of the previous studies conducted in the area have relied on relatively few days of repeated observations, mainly seven consecutive days [20], when calculating their predictions, although there are some notable exceptions [6, 14, 19].

The aim of the present study is to estimate the length of time an individual needs to be monitored using accelerometers in order to estimate the usual physical activity level to a desired level of precision.

## Methods

### Study population

The study used a convenience sample consisting of university students and staff as well as staff recruited from nearby worksites. The participants were contacted by e-mail. They were sent information regarding the nature of the study and what was expected of them. If they were interested in participating in the study they were asked to reply to the e-mail.

To capture more of the natural variation in physical activity than is typically done in studies, a four-week protocol instead of the standard seven-day one was used. The participants were asked to wear the accelerometer during waking hours, only taking it off during water-based activities and while sleeping. The participants received a total of three visits from a member of the research group. At the first visit, the participants were instructed on how to properly position the accelerometer at the right hip and they got a brief explanation of how the accelerometer works. They were also instructed that they could at any time without explanation leave the study and that the research group promised to keep all information that was collected during the study confidential. The second visit took place approximately two weeks after the first visit, during which the batteries of the accelerometer were charged. This took approximately two hours. The third visit took place after an additional two weeks and at this point the accelerometer was returned and the participants were asked to complete a questionnaire where socio-demographic and brief health information was gathered. Informed consent was obtained from all participants and the study was approved by the regional ethical committee in Linköping, Sweden (Dnr: 2016/30-31).

### Assessment of physical activity

Two different accelerometers were used (Actigraph models GT1M or GT3X; ActiGraph. LLC Pensacola. FL) which have been shown to provide comparable outputs using the vertical axis [23]. Therefore, only data from the vertical axis of the accelerometer was used in this study.

The accelerometer was set to collect data using 5-second epochs. After data collection, the data was treated according to a commonly used data cleaning procedure: for a day to be considered as valid, the wear time had to exceed >600 min * day^-1^ once periods of >20 minutes of consecutive epochs with 0 counts had been removed. Only those with at least 21 days of valid monitoring were included in the subsequent analysis. To calculate the duration of physical activity at different intensities the following cut-points were used: <100 counts per minute (cpm) for sedentary behaviour [24], 100–1951 cpm for light physical activity, 1952–5723 cpm for moderate physical activity, 5724 and higher for vigorous physical activity [25]. "At least moderate intensity physical activity" (MVPA) was calculated as the sum of all epochs with 1952 cpm or more (i.e. moderate + vigorous).

### Data handling and statistics

To be able to compare the level of physical activity within different groups, the variables derived from the questionnaire were recoded so that age was divided into two categories according to the median: younger than 31 years or 31 years or older. Body Mass Index (BMI) was calculated as self-reported weight divided by height squared $\frac{kg}{m^{2}}$ and divided into two categories: under- or normal weight ≤25 $\frac{kg}{m^{2}}$ and overweight/obese >25 $\frac{kg}{m^{2}}$. The self-rated health variable was recoded from five categories (excellent, very good, good, somewhat or poor) to two, (excellent and very good versus the other three). The number of valid days the accelerometer was worn was divided according to the median; ≤ 27 days or 28 days or more. Occupational physical activity was recoded from three categories; mostly sitting, sitting and standing and heavy manual labour to two categories; sedentary which includes the mostly sitting and non-sedentary which includes the other two. The highest level of education was recoded from originally five categories into two: having or not having an education at university level. Bootstrapped mean values and 95% confidence intervals (95% CI) around the mean were calculated for all physical activity variables, stratified by the socio-demographic variables. To investigate if there was any systematic difference in the levels of physical activity between the different socio-demographic variables, an independent samples t-test was performed. The analysis was conducted using IBM SPSS Statistics v 23 (IBM SPSS Statistics for Windows. Armonk. NY: IBM Corp.).

To estimate the number of repeated observations that are needed to calculate the habitual level of physical activity to a given level of precision, the following procedure was used.

First, the within-subject variation, expressed as the within-subject coefficient of variation in percentage (CVw) was calculated according to:

$$CVw=(\frac{SDw}{\overset{-}{x}})*100$$ (1)

in which SD_w_ is the within-subject standard deviation and $\overset{-}{x}$ is the mean of the individual. The estimates of the within-subject coefficient of variation (CVw) were bootstrapped 2000 times within each individual and each bootstrapped estimate was saved. Each of the saved estimates was then entered in Eq 2. By bootstrapping the estimates, it is possible to assign measures of accuracy, such as confidence intervals, to the sample estimates [26]. Thus, the distribution around the point estimates can be presented, which gives an idea of the certainty of the estimates.

Secondly, to calculate the number of repeated observations needed to estimate, to a given level of precision, the usual physical activity of an individual, the following formula was used [4, 8]

$$D={\left(\frac{Z\alpha *CVw}{D_{0}}\right)}^{2}$$ (2)

In which D is the number of days needed to monitor. Zα is the normal deviate for which the percentage of time the measured value should fall within a specified limit (i.e 1.96 = 95% confidence, 1.28 = 80%). CVw is the within-subject coefficient of variation obtained from the bootstrapped estimates previously described, and D_0_ is the desired precision (e.g. 20%) within the habitual level of physical activity which the observed level should fall. The outcome from such an analysis is interpreted as the number of repeated observations needed to be able to say, with a given level of precision, that the observed level of habitual physical activity falls within the specified limit of the level of habitual physical activity. In this study, the number of days needed to monitor an individual in order to be within 5–50% (D_0_ = 5–50) of the level of habitual physical activity 95% (Zα = 1.96) of the time was calculated.

The number of days required to monitor a group of individuals so that between 50% and 95% of the sample would be within 5–50% of their habitual level of physical activity was also calculated.

To investigate if there was any systematic association between the level of physical activity and CVw, a linear regression model between CVw and duration of physical activity at the different intensity categories was fitted and presented graphically. The analysis was conducted in R version 3.2.0 [27] and the graphs were produced using the package ggplot2 [28].

## Results

Out of the 61 subjects who initially volunteered, 50 provided valid data, i.e. at least 21 days of physical activity data. There was no difference in any of the physical activity variables between those that provided valid information and those that did not, (independent samples t-test all p>0.05). Men were more sedentary compared to women (p = 0.038), those with more valid days of monitoring were more physically active on a light intensity (p = 0.005) and moderate intensity level (p = 0.024) and accumulated more time at MVPA (p = 0.014) compared to those with fewer days of monitoring (Table 1). Subjects with a sedentary occupation accumulated more time in sedentary activity compared to those that had a non-sedentary occupation (p = 0.043).

**Table 1:** Descriptive data of the studied population. The 95% CI is based on bootstrapped estimates (k = 2000) and the p-values are from an independent samples t-test.

	N	Sedentary			Light			Moderate			Vigorous			MVPA		
		Mean	95% CI	p	Mean	95% CI	p	Mean	95% CI	p	Mean	95% CI	p	Mean	95% CI	p
Sex				0.038			0.137			0.632			0.227			0.451
Men	15	673	654–693		142	128–157		55	46–67		8	6–11		63	53–75	
Women	31	645	626–661		127	115–141		52	45–60		6	4–8		58	49–67	
Age				0.274			0.381			0.237			0.364			0.195
Under 31	23	660	646–674		127	114–141		57	49–64		8	5–10		64	56–72	
31 or older	27	646	625–667		135	122–148		50	42–58		6	3–9		56	47–66	
Days measured				0.788			0.005			0.024			0.07			0.014
Up to 27	21	650	626–671		116	103–130		46	38–52		5	3–7		50	42–58	
28 or higher	29	654	638–670		143	132–154		58	51–66		8	6–11		66	58–75	
Body Mass Index				0.790			0.387			0.483			0.45			0.400
Normal	26	654	640–668		127	116–139		55	47–63		7	5–10		62	54–71	
Overweight/obese	24	651	626–672		136	121–150		51	42–59		6	4–8		57	47–66	
Health				0.676			0.280			0.912			0.373			0.740
Very good or excellent	26	655	634–673		136	124–149		53	46–61		7	5–10		61	52–70	
Poor to good	24	650	633–667		126	112–140		53	44–61		6	3–9		58	49–67	
Occupational PA				0.043			0.077			0.099						0.065
Sedentary	20	668	653–682		121	107–136		59	50–68		8	5–12		67	58–76	
Non sedentary	30	642	624–661		138	127–150		49	41–57		6	4–7		55	46–63	
Education				0.097			0.128			0.212			0.169			0.133
Non-university	19	638	610–664		140	124–158		48	38–59		5	3–8		53	41–65	
University	31	631	648–673		126	115–136		56	50–63		8	5–8		64	57–71	
TOTAL	50	652	639–665		131	122–141		53	47–59		7	5–9		60	53–66	

**Fig 1:**
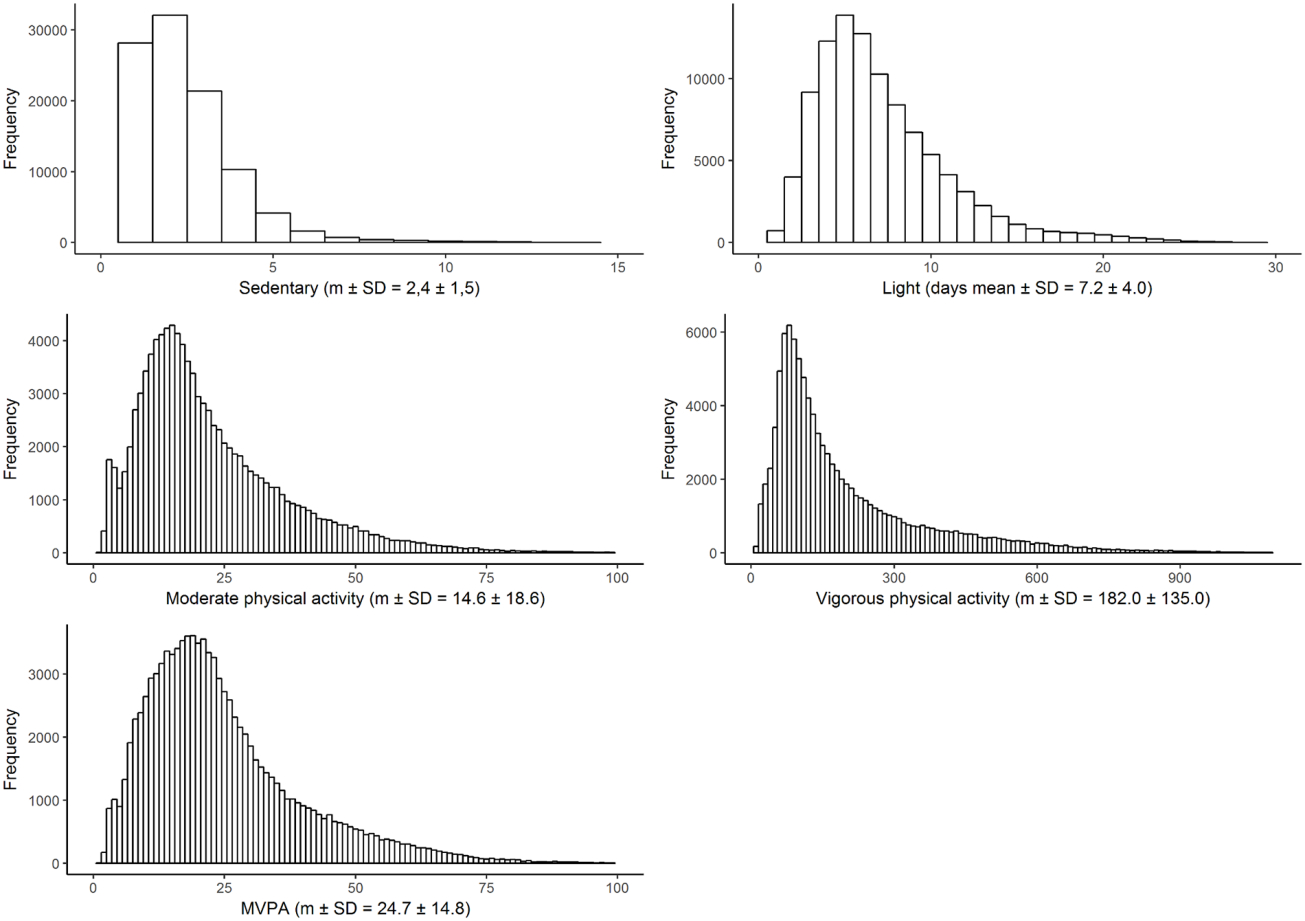
Histograms depicting the bootstrapped (k = 2000) estimates of number of days needed to with 95% confidence be within 20% of the habitual level of physical activity of an individual at different intensities.

Histograms depicting the number of days needed to with 95% confidence be within 20% of the habitual physical activity of an individual at different intensities is shown in Fig 1. The mean number of days needed is highest for vigorous physical activity in which 182 days are needed. For sedentary behaviour the equivalent number of days is 2.4 days.

The number of days required to monitor the studied sample so that the physical activity of between 50% and 95% of the sample is within 5–50% of their habitual level is shown in Table 2. To capture 80% of the sample’s habitual level of physical activity to a precision of ± 20% at different intensities 3.4 days is needed if sedentary behavior is the outcome of interest, 9.8 days for light intensity physical activity, 32.5 days for moderate intensity physical activity, 302.2 days for vigorous intensity physical activity, and 34.8 days for MVPA.

**Table 2:** Based on the bootstrapped estimates, the number of days that are required to identify between 50% and 95% of the sample within 5–50% of their habitual level of physical activity is shown. E.g. to capture the sedentary activity of at least 80% of the sample to a level of precision of ±20% of their habitual level of sedentary behavior, 3.4 days of monitoring is needed. Mean refers to the estimated mean level of habitual physical activity based on the within-subject variation.

	Percentage of the study sample						
	50%	60%	70%	75%	80%	90%	95%
Sedentary							
Mean ± 5%	34.1	39.6	45.8	49.5	53.9	66.7	79.9
Mean ± 10%	8.5	9.9	11.5	12.4	13.5	16.7	20.0
Mean ± 15%	3.8	4.4	5.1	5.5	6.0	7.4	8.9
Mean ± 20%	2.1	2.5	2.9	3.1	3.4	4.2	5.0
Mean ± 30%	0.9	1.1	1.3	1.4	1.5	1.9	2.2
Mean ± 40%	0.5	0.6	0.7	0.8	0.8	1.0	1.2
Mean ± 50%	0.3	0.4	0.5	0.5	0.5	0.7	0.8
Light intensity physical activity							
Mean ± 5%	100.3	114.8	133.0	144.0	157.1	195.8	237.2
Mean ± 10%	25.1	28.7	33.2	36.0	39.3	48.9	59.3
Mean ± 15%	11.1	12.8	14.8	16.0	17.5	21.8	26.4
Mean ± 20%	6.3	7.2	8.3	9.0	9.8	12.2	14.8
Mean ± 30%	2.8	3.2	3.7	4.0	4.4	5.4	6.6
Mean ± 40%	1.6	1.8	2.1	2.3	2.5	3.1	3.7
Mean ± 50%	1.0	1.1	1.3	1.4	1.6	2.0	2.4
Moderate intensity physical activity							
Mean ± 5%	297.3	350.8	421.5	466.0	519.7	678.4	826.1
Mean ± 10%	74.3	87.7	105.4	116.5	129.9	169.6	206.5
Mean ± 15%	33.0	39.0	46.8	51.8	57.7	75.4	91.8
Mean ± 20%	18.6	21.9	26.3	29.1	32.5	42.4	51.6
Mean ± 30%	8.3	9.7	11.7	12.9	14.4	18.8	22.9
Mean ± 40%	4.6	5.5	6.6	7.3	8.1	10.6	12.9
Mean ± 50%	3.0	3.5	4.2	4.7	5.2	6.8	8.3
Vigorous intensity physical activity							
Mean ± 5%	2160.5	2711.8	3526.3	4094.2	4835.9	7187.1	9185.0
Mean ± 10%	540.1	677.9	881.6	1023.5	1209.0	1796.8	2296.2
Mean ± 15%	240.1	301.3	391.8	454.9	537.3	798.6	1020.6
Mean ± 20%	135.0	169.5	220.4	255.9	302.2	449.2	574.1
Mean ± 30%	60.0	75.3	98.0	113.7	134.3	199.6	255.1
Mean ± 40%	33.8	42.4	55.1	64.0	75.6	112.3	143.5
Mean ± 50%	21.6	27.1	35.3	40.9	48.4	71.9	91.8
MVPA							
Mean ± 5%	341.6	392.0	457.7	500.7	556.7	729.8	879.1
Mean ± 10%	85.4	98.0	114.4	125.2	139.2	182.4	219.8
Mean ± 15%	38.0	43.6	50.9	55.6	61.9	81.1	97.7
Mean ± 20%	21.4	24.5	28.6	31.3	34.8	45.6	54.9
Mean ± 30%	9.5	10.9	12.7	13.9	15.5	20.3	24.4
Mean ± 40%	5.3	6.1	7.2	7.8	8.7	11.4	13.7
Mean ± 50%	3.4	3.9	4.6	5.0	5.6	7.3	8.8

**Fig 2:**
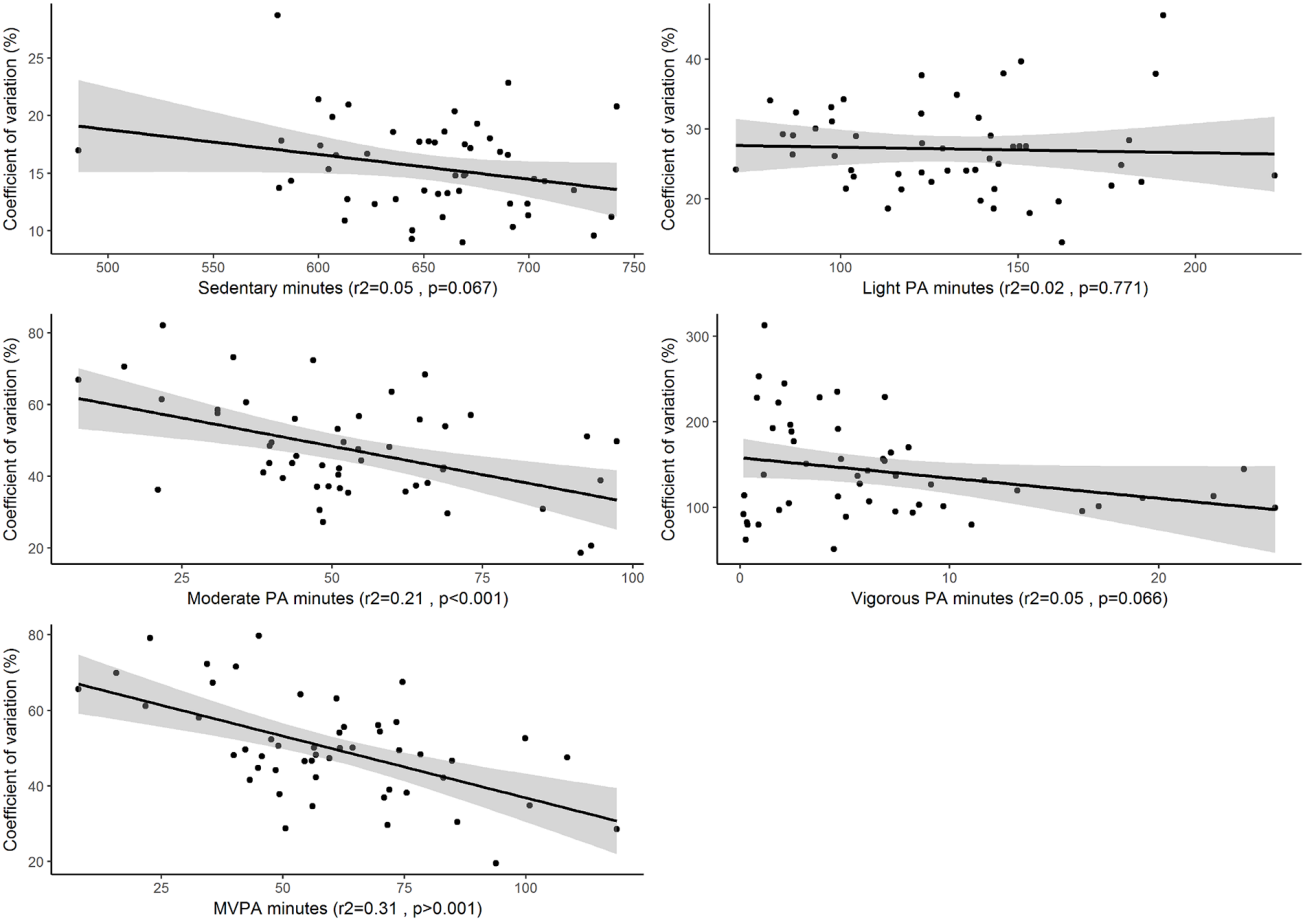
The association between the amount of physical activity at different intensities and the CVw as illustrated by a scatter plot with the regression line and its 95% CI (the shaded area).

In general, the mean level of time spent in the different intensity levels had small effects on the within-subject coefficient of variation (Fig 2). For moderate intensity physical activity (R^2^ = 0.21, p<0.001) as well as MVPA (R^2^ = 0.31, p<0.001), a negative slope was observed, indicating that the more time spent at those intensities, the lower the coefficient of variation is.

**Fig 3:**
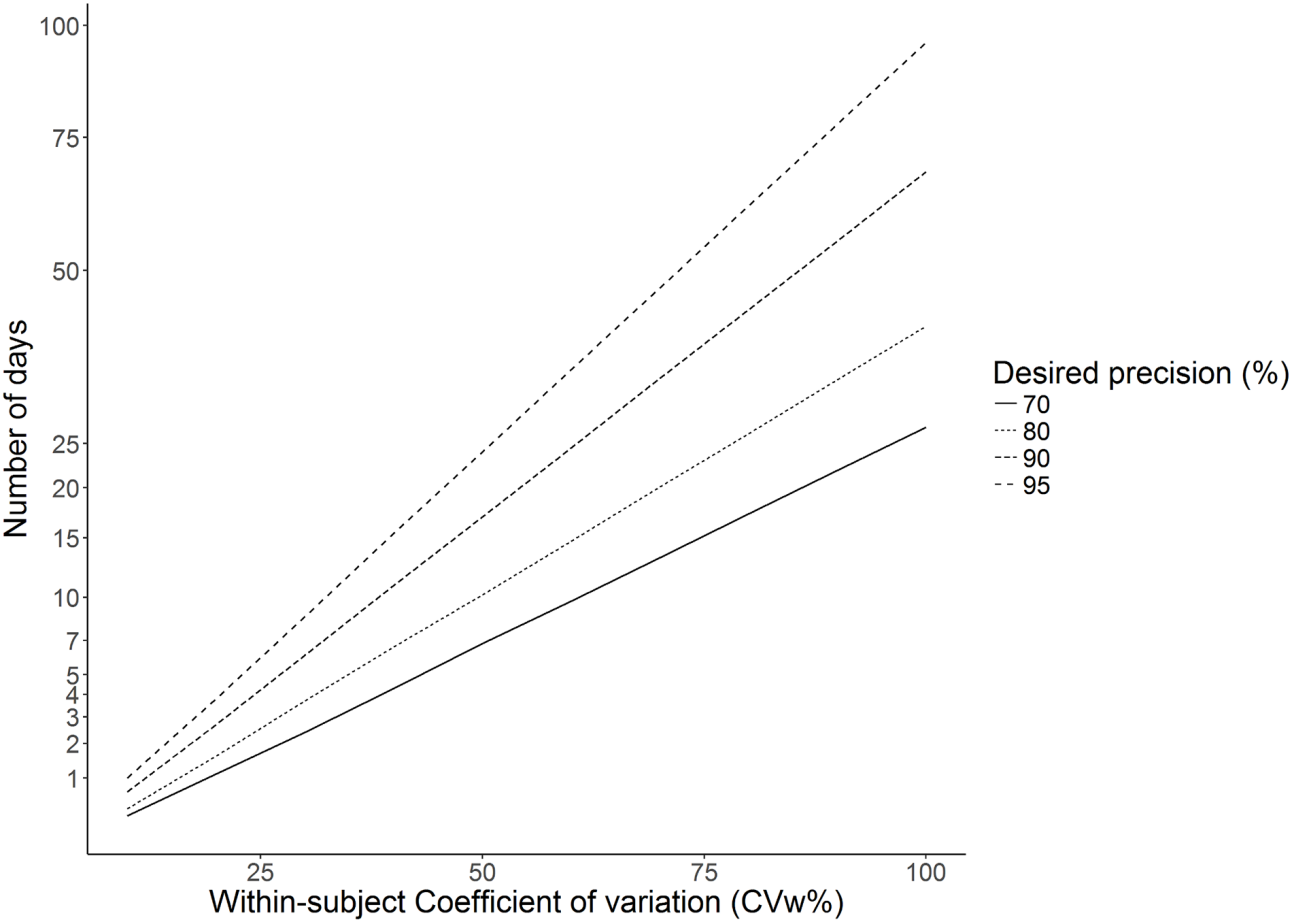
The theoretical number of days needed to monitor an individual, assuming a within-subject coefficient of variation (CVw) of between 10% and 100% to be within ± 20% of the level of an individual’s habitual physical activity 70–95% of the time.

The number of days required to monitor an individual to estimate his or her level of habitual physical activity varies by the desired level of precision and/or the CVw. In Fig 3 the theoretical number of days needed to monitor an individual, assuming within-subject coefficients of variation (CVw) of between 10% and 100% to be within ± 20% of the level of an individual’s habitual physical activity 70–95% of the time is illustrated.

## Discussion

To date, there is no method that can assess physical activity behaviour without measurement error and therefore it is important to know the size of the measurement error particularly when planning studies. Depending on what kind of analysis is planned, and therefore the level of precision that is desired, the number of subjects or of repeated observations within subjects, or both, needs to be correctly estimated. In this study, the number of days needed in order to identify the habitual physical activity to a given level precision in individuals was calculated.

This study indicates that the widely used protocol of measuring physical activity with accelerometers for seven days may be too short a period if the aim is to perform correlation or regression analysis at individual rather than group level. Using the 7-day monitoring period, one can be confident that the level of sedentary activity observed is within ±15% of the habitual level 95% of the time for more than 80% of the observations. However, in this study ± 15% corresponds to between 558 and 750 minutes, a very large time span. For MVPA the standard protocol of seven days of measurement produced an outcome in which the 80th percentile of number of days needed was only observed if the precision was within 50% of the habitual level. Given that the observed mean level of MVPA in this study was 60 minutes, one can expect that the habitual activity level lies between 30–90 minutes 95% of the time, for 80% of the sample. For the other 20% the mean will fall outside this interval. This calculation illustrates how difficult it is to identify an individual's habitual physical activity level at higher intensities and it also illustrates that results from interventions that are not expected to have a very large effect will probably appear non-significant due to the very large background noise from the natural variation in physical activity behaviour which leads to regression attenuation [29]. Attenuated correlation or regression coefficients between physical activity and a health outcome will bias the result towards null, or even become non-significant. This may lead to important associations being ignored.

The results of the present study confirm, to some extent, the previous studies that have estimated the number of days to rank individuals [10–22]. Those studies have also observed that vigorous physical activity is the physical activity behavior by which it is most difficult to reliably rank individuals [12]. One possible reason why vigorous physical activity is so difficult to determine with any real precision, both in terms of ranking as well as the absolute level of habitual behaviour, is simply because it is such a sporadic behaviour. Even if the mean level of vigorous physical activity in our study was 7 minutes (95% CI 5–9) (Table 1), the vast majority of individuals accumulated less than 10 minutes of vigorous physical activity per day on average. The usual sedentary behaviour was the behaviour most easily to determine on an individual level in this study. However, some studies have shown that sedentary behaviour is not always the behaviour that requires the fewest days of observation to rank individuals [6, 10, 14, 17]. The difference between the habitual sedentary behaviour and the ranking of individuals is most likely due to the fact that the within-subject variation is small in terms of absolute numbers but relatively large in relation to the between-subject variation. This will lead to a small ICC and a higher number of observations needed to rank individuals. The small within-subject variation illustrates a stable behaviour, thus fewer days are required to identify the habitual physical activity behaviour. The sedentary behaviour is also “researcher dependent”, i.e. it depends on the choice made when defining non-wear time. Mâsse, L et al compared four different algorithms of which one was the same as in the present study [30]. The data from that study show that the algorithm used in the present paper produced the lowest CV leading to the fewest days needed to identify the usual sedentary behavior of an individual. Future studies should further investigate the influence on wear-time definitions on the precision of the outcome.

Another observation was that the CVw was lower among those with high levels of physical activity, particularly for MVPA. This illustrates another point that researchers should be aware of, namely that subjects with the lowest levels of MVPA, for whom most interventions are designed, may be those whose habitual physical activity behaviour is the most difficult to identify. Based on the information in Fig 2 the average CVw for those that accumulate the least MVPA is around 70% while in the upper end of the amount of MVPA accumulated the CVw is around 30%.

The outcome of the present study presents some challenges for studies that need to collect accurate data on individual level (i.e. level 4). For these studies a longer measurement period may be needed, resulting in an increase in participant burden for the subjects as well as to increased study costs due to a slower turn-around rate of the accelerometers. However, a longer measurement period increases the precision of the measurement which means that fewer subjects are needed. Ideally, the number of days required for a specific study should be estimated from a small pilot study or from previous studies on the relevant population. Failing that, figures based on simulations such as those presented here can be used. Secondly this study illustrates that the conventional way of analysing accelerometer data, i.e. calculating how much time an individual has accumulated at different intensities may not be the most realistic method if the habitual level of physical activity is of interest. In any given sample there are differences in for example body mass index (BMI) which influence the relative intensity an individual’s count-value corresponds to [31]. The use of absolute cut-points does not consider this. A few promising attempts to circumvent this, by investigating the shape of the count distribution rather than the accumulated sum of all epochs with counts above certain thresholds, has been conducted [32, 33]. This procedure may be a way forward but still requires a lot of testing before any solid conclusions can be drawn.

### Limitations and strengths

The major limitation of this study is the small sample size and that the sample was not selected at random. This limits the generalisability of the study. Furthermore, the study sample was on average a relatively active sample, which also limits the generalisability of the findings. Another limitation is that only the vertical axis of the accelerometer was used. Using the vertical axis alone may result in some physical activity being missed, however research shows that most of the information regarding physical activity is carried in the vertical axis and including the other two to estimate a vector magnitude does not increase the validity considerably [34]. There are also other factors that need to be considered when designing a study, which were not investigated in this study but which future studies should investigate, such as the effect of different data cleaning procedures, the cost-benefit trade-off of longer measurement periods vs a larger study sample.

The major strength of this study is that, compared to most other studies, a long assessment period was used. A longer period will allow capture more of the normal day-to-day variation of physical activity, thus provide a better estimate of the usual physical activity behaviour of an individual. Previous research using accelerometers has predominantly used a 7-day protocol, although there are exceptions [14, 15]. A longer measurement period may also reduce the risk of the "Hawthorne effect", i.e. that the subjects will behave differently when they know that they are being monitored. Another of the strengths is that the different statistical estimates were bootstrapped. This gives an estimation of the population distribution based on the sample distribution, which in theory increases the generalisability of the study.

### Conclusion

The present study shows that for analyses requiring accurate data at the individual level a longer measurement collection period than the traditional 7-day protocol should be used. In addition, the amount of MVPA was negatively associated with the number of days required to identify the habitual physical activity level indicating that the least active are those whose habitual physical activity levels are the most difficult to identify.

These results could have important implications for researchers whose aim is to collect and analyse data on individual level. Before recommendations regarding an appropriate monitoring protocol are updated, the present study should be replicated in different populations.
